# A comprehensive evaluation of adaptive daily planning for cervical cancer HDR brachytherapy

**DOI:** 10.1120/jacmp.v17i6.6408

**Published:** 2016-11-08

**Authors:** Rebecca Meerschaert, Adrian Nalichowski, Jay Burmeister, Arun Paul, Steven Miller, Zhenghui Hu, Ling Zhuang

**Affiliations:** ^1^ Department of Oncology Radiation Oncology Division Wayne State University School of Medicine Detroit MI USA; ^2^ Department of Oncology Radiation Oncology Division Karmanos Cancer Institute Detroit MI USA; ^3^ Center for Optics and Optoelectronics Research College of Science Zhejiang University of Technology Hangzhou Zhejiang China

**Keywords:** cervical cancer, adaptive daily plan, interfractional organ variation, HDR

## Abstract

The purpose of this study was to evaluate adaptive daily planning for cervical cancer patients who underwent high‐dose‐rate intracavitary brachytherapy (HDR‐BT) using comprehensive interfractional organ motion measurements. This study included 22 cervical cancer patients who underwent 5 fractions of HDR‐BT. Regions of interest (ROIs) including high‐risk clinical tumor volume (HR‐CTV) and organs at risk (OARs) were manually contoured on daily CT images. All patients were clinically treated with adaptive daily plans (ADP), which involved ROI delineation and dose optimization at each treatment fraction. Single treatment plans (SP) were retrospectively generated by applying the first treatment fraction's dwell times adjusted for decay and dwell positions of the applicator to subsequent treatment fractions. Various existing similarity metrics were calculated for the ROIs to quantify interfractional organ variations. A novel similarity (JRARM) score was established, which combined both volumetric overlap metrics (DSC, JSC, and RVD) and distance metrics (ASD, MSD, and RMSD). Linear regression was performed to determine a relationship between interfractional organ variations of various similarity metrics and $D_{2\text{cc}}$ variations from both plans. Wilcoxon signed‐rank tests were used to assess ADP and SP by comparing $\text{EQD}2\;D_{2\text{cc}}(\alpha /\beta =3)$ for OARs. For interfractional organ variations, the sigmoid demonstrated the greatest variations based on the JRARM, DSC, and RMSD metrics. Comparisons between paired ROIs showed differences in metrics at each treatment fraction. RVD, MSD, and RMSD were found to be significantly correlated to $D_{2\text{cc}}$ variations for bladder and sigmoid. The comparison between plans found ADP provided lower EQD2 $D_{2\text{cc}}$ of OARs than SP. Specifically, the sigmoid demonstrated statistically significant dose variations (p=0.015). Substantial interfractional organ motion occurs during HDR‐BT based on comprehensive measurements and may significantly affect $D_{2\text{cc}}$ of OARs. Adaptive daily planning provides improved dose sparing for OARs compared to single planning with the extent of sparing being different among OARs.

PACS number(s): 87.55.D, 87.55.de, 87.55.kh, 87.57.nj

## I. INTRODUCTION

Cervical cancer is the third most prevalent cancer in females worldwide.^(1)^ The current standard of care for locally advanced cervical cancer is a combination of external beam radiotherapy (EBRT) and intracavitary brachytherapy (BT) with concurrent chemotherapy, providing high rates of local disease control.^2^, ^3^ High‐dose‐rate (HDR) BT is an important component in the curative management of cervix carcinoma and BT is typically delivered in 4 to 6 fractions using ring and tandem (R+T) or tandem and ovoid (T+O) applicators.^(4)^ A single plan (SP) approach for HDR treatments involves contouring and treatment planning at the first fraction and applying the treatment plan to remaining treatment fractions. However, this approach does not take into account interfractional applicator positioning variations and organ motion that may lead to substantial differences between planned and delivered doses.^5^, ^6^, ^7^, ^8^, ^9^

Previous studies have shown that interfractional organ motion in the pelvis may be substantial.^10^, ^11^, ^12^, ^13^, ^14^, ^15^, ^16^, ^17^ Lee et al.^(15)^ found average changes in cervix position can be up to 10 mm, 8 mm, and 16 mm in the lateral, superior/inferior, and anterior/posterior directions, respectively. Such variations in interfractional applicator positioning and organ motion have been shown to result in significant differences between planned and delivered dose.^5^, ^6^, ^9^ Chakraborty et al.^(5)^ found that 47% of rectal and 19% of bladder dose variations resulted from applicator shifts between treatment fractions and the remaining variations were a result of organ variations. This relation between interfractional organ motion and dose variation has been studied for variations in organ volume and organ distance‐to‐applicator with respect to variations in mean organ dose, reference point dose, and volume dose.^(6,16,18–20)^ However, a comprehensive method to analyze the relationship between interfractional organ and dose variations that takes into account both volumetric and organ displacement variations is lacking. While many of these studies only investigated bladder and rectum as organs at risk (OARs), other studies have determined the importance of the sigmoid as an OAR.^3^, ^21^ A significant relationship has been found between sigmoid‐to‐tandem distance and sigmoid dose, in addition to the sigmoid receiving an excess of 70% of the intended point A dose; therefore, it is essential to include the sigmoid as a critical organ.^3^, ^21^

Adaptive planning has been implemented by various institutions for cervical cancer HDR‐BT. An adaptive daily plan (ADP) approach for HDR treatments involves contouring and treatment planning at each fraction. Research involving adaptive planning has been implemented using daily MR images.^(9,22–24)^ Due to the lack of MRI scanner availability in most Radiation Oncology departments, along with the MRI distortion issue, MR scanner availability and staff resources, this method lacks wide use.^25^, ^26^ Limited research has been done on CT imaging based adaptive daily planning,^8^, ^27^ therefore, in this study, ADP was further evaluated by performing a plan comparison with SP.

In this study, we fully investigated CT‐based adaptive daily planning by first investigating the interfractional variations of HR‐CTV, bladder, rectum, and sigmoid contours during HDR‐BT through comprehensive measures involving volumetric and distance similarity metrics. These interfractional variations were then studied to determine their effect on dose variations. Additionally, the ADP created at each HDR fraction was retrospectively compared with the simulated SP to evaluate the dose delivered to the HR‐CTV and OARs.

## II. MATERIALS AND METHODS

### A. Patient population

This study included 22 cervical cancer patients treated with HDR‐BT between March 2011 and March 2015 at the Karmanos Cancer Center in Detroit, Michigan. All patients had biopsy‐proven uterine cervical cancer (stage IB‐IVA) and were administered definitive radiotherapy with concurrent chemotherapy (Cisplatin). Radiotherapy involved EBRT, with a total dose of 37.8 to 45.0 Gy, and consecutive HDR‐BT. All patients underwent 5 fractions of HDR treatments with consistent R+T applicator size and a prescription dose of 5 to 6 Gy to Point A or modified Point A at each fraction. Complete treatment fractionation schemes are listed in Table 1.

**Table 1:** Number of patients for each HDR‐BT fractionation scheme

*Treatment Fractionation Scheme*	*Frequency*
5.50Gy×5fx	11
6.00Gy×3fx; 5.50Gy×2fx	3
6.00Gy×4fx; 5.50Gy×1fx	1
5.50Gy×4fx; 5.00Gy×1fx	1
6.00Gy×2fx; 5.50Gy×3fx	1
5.50Gy×4fx; 5.25Gy×1fx	1
5.50Gy×3fx; 5.00Gy×2fx	1
5.00Gy×4fx; 5.50Gy×1fx	1
5.75Gy×1fx; 5.50Gy×3fx; 5.25Gy×1fx	1
6.00Gy×2fx; 5.50Gy×2fx; 5.00Gy×1fx	1

fx=fraction.

### B. Image data and organ delineation

**Figure 1:**
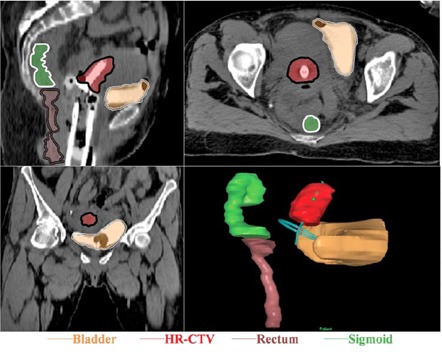
CT image data for the delineated regions of interest as displayed in the planning system and the three‐dimensional view of the contours.

A static CT image was acquired with a Somatom CT scanner (Siemens, Erlangen, Germany) prior to each HDR fraction with an in‐plane image size of 512×512 pixels, slice thickness of 3 mm, and in‐plane image resolution of approximately 1 mm. Regions of interest (ROIs) including the HR‐CTV, bladder, rectum, and sigmoid were manually contoured on each CT image by physicians. The HR‐CTV incorporated the gross tumor volume and the entire uterine cervix.^(28)^ Figure 1 shows three orthogonal views of example CT data with ROI contours and the resulting three‐dimensional view in the planning system.

**Figure 2:**
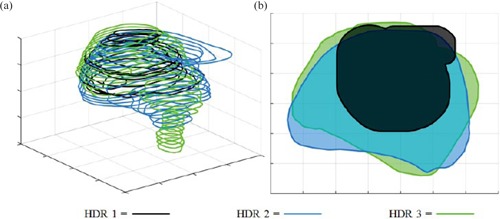
Rectum contours only for HDR fractions 1–3 for visualization purposes. Contours are all mapped to the coordinate of CT1 (a) in a three‐dimensional view and (b) in a two‐dimensional view by taking a cross section.

The planning CT image from the first HDR treatment (CT1) was used as the reference image to which CT images from the remaining treatment fractions for patients with same applicator geometry were rigidly registered using in the Eclipse treatment planning system (Varian Medical Systems, Palo Alto, CA). The rigid registration refers to bony anatomy alignment as investigated by Elhanafy et al.^(6)^ Contours of ROIs were mapped to the coordinates of CT1 to calculate similarity metrics (see Materials and Methods section D). MATLAB (MathWorks, Natick, MA) was used to develop a program for contour similarity calculations (details in section D below). Figure 2 shows the contours of the rectum for a sample patient, where all contours are mapped to the coordinates of CT1. Only contours from fractions 1 to 3 are displayed here for visualization simplification.

### C. Treatment planning

All patients in this study were treated using adaptive daily HDR‐BT treatment plans. For treatment planning, dose optimization was based on applicator positioning in the Oncentra Brachytherapy treatment planning system (Nucletron, Veenendaal, The Netherlands). Prior to each treatment, a Foley catheter was inserted, which remained during planning CT imaging and HDR‐BT and was unclamped between imaging and treatment. Saline was injected into the bladder prior to imaging and treatment to maintain more consistent volumes throughout all treatment fractions. Preceding each treatment delivery, ROIs were manually contoured on the planning CT image and assigned the prescription dose to Point A or modified Point A while trying to maintain the bladder $D_{2\text{cc}}<90\;{\text{Gy}}_{\text{EQD}2}$ and the rectum and sigmoid $D_{2\text{cc}}<75\;{\text{Gy}}_{\text{EQD}2}$ for combined EBRT and HDR‐BT.^(29)^ The dose distribution was then modified by physicists to achieve the best normal tissue sparing. For plan comparison, SP were retrospectively generated for each patient for treatment fractions 2 through 5 by applying the dwell times and positions from the first fraction to the applicator locations in each subsequent fraction. The dwell times were rescaled by the ratio of source strength in the prescription compared to treatment data to account for source decay.

### D. Organ contour evaluation measures and statistical analysis

Conventional contour similarity measures fall into two categories: volumetric overlap measurements such as Dice similarity coefficient (DSC),^(28)^ Jaccard similarity coefficient (JSC),^(30)^ and relative volume difference (RVD),^(31)^ and distance measurements including average symmetric absolute symmetric surface distance (ASD),^(32)^ root mean square symmetric absolute surface distance (RMSD),^(32)^ and maximum symmetric absolute surface distance (MSD).^(32)^ When comparing contours using existing similarity measures, conflicting measures may lead to difficult interpretation. For example, for the bladder having similar MSDs around 21 mm, the corresponding DSCs were vastly different at 0.75 and 0.52. Here we propose a single measurement that embodies the JSC, RVD, ASD, RMSD, and MSD parameters to provide a comprehensive understanding of organ variation by incorporating both volumetric overlap and distance metrics using the following equation:

$$\begin{array}{l} JRARM\;score=w_{1}f_{1}(JSC)+w_{2}f_{2}(RVD)+w_{3}f_{3}(ASD)+w_{4}f_{4}(RMSD)+\\ \;w_{5}f_{5}(MSD) \end{array}$$

where $w_{i}$ corresponds to the weight given to the function $f_{i}$ (ranging from 0 to 100) of each metric. JRARM score provides a single similarity metric for overall similarity evaluation leading to less confusion. For simplicity, $w_{i}(i=1$ to 5) was chosen to be equal (0.2) for each metric $f_{i}(i=1$ to 5) to scale JRARM between 0 and 100 in this study. Since DSC and RMSD are well‐known metrics for assessing volume overlap and displacement, they were used for contour comparison in addition to JRARM for this study.

For each patient, the JRARM score, DSC, and RMSD were calculated for HDR fraction i (i=2 to 5) to measure each ROI's contour similarity from reference fraction 1(HDRi‐1(i=2 to 5)). Interfractional organ variations were defined by organ overlap and organ displacement based on registered bony pelvic anatomy and were investigated through comparing the calculated JRARM score, DSC, and RMSD among HDR fraction i(i=2 to 5) using box plots.

### E. Dose evaluation measures and statistical analysis

Dose‐volume histograms (DVHs) for the bladder, rectum, sigmoid, and HR‐CTV were exported from the Oncentra Brachytherapy treatment planning system for ADP and SP. The dose received by most irradiated 2 cubic centimeters ($D_{2\text{cc}}$) and $D_{0.1\text{cc}}$ were reported for each OAR and dose received by 90% of the volume of interest ($D_{90}$) was reported for the HR‐CTV.^(9)^ MATLAB was used to calculate these dose parameters ($D_{0.1\text{cc}},D_{2\text{cc}}$, and $D_{90}$) for each plan from the cumulative DVHs. $D_{2\text{cc}}$ and $D_{90}$ were used to calculate physical dose variations, defined as the ratio of mean dose change between fractions to mean dose of the first fraction, as described previously, to quantify variations from the initial plan.^(23)^ Physical doses were converted to biological doses through the linear‐quadratic model for sublethal cell damage repair with the tissue parameter value α/β=3Gy for OARs and α/β=10Gy for HR‐CTV.^(33)^ In addition, sparing factors, which are defined as a ratio of $D_{2\text{cc}}$ for a specific OAR to $D_{90}$ for the HR‐CTV per HDR fraction, as described previously, were used for each OAR for plan comparison.^34^, ^35^ Sparing factors provided a metric to compare planning methods that took into account both OAR dose and HR‐CTV dose where lower sparing factors correspond to more favorable outcomes.

Linear regression analysis was performed to determine the relationship between interfractional organ variations, as described by the similarity metrics, and the interfractional dose variations, as described by $D_{2\text{cc}}$ variations. Wilcoxon signed‐rank tests were conducted to compare $D_{2\text{cc}}$ from the single plan to the adaptive daily plan for each OAR's total HDR‐BT biological dose. Any p‐value less than 0.05 was taken to be significantly different for statistical tests. Descriptive statistics and Wilcoxon signed‐rank tests were acquired using SPSS (IBM, Armonk, NY).

## III. RESULTS

### A. Interfractional organ and dose variations

**Figure 3:**
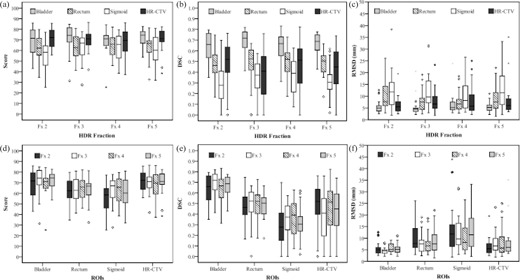
Boxplots showing organ variation from fx 1 for all fx of each ROI by comparing (a) JRARM scores, (b) DSCs, and (c) RMSDs in addition all ROI within each fx by comparing (d) JRARM scores, (e) DSCs, and (f) RMSDs. Fx=HDR fraction, circles represent outliers (between 1.5 and 3 interquartile ranges beyond first or third quartile), and ^*^ represent extreme outliers (more than 3 interquartile ranges beyond first or third quartile).

Table 2 shows the mean and standard deviation for DSCs and JRARM scores calculated from Eq. (1). The bladder has the highest DSC and JRARM score throughout fractions, whereas the sigmoid has the lowest. The sigmoid had the greatest variation in JRARM scores throughout the course of treatment, whereas the bladder, rectum, and HR‐CTV remain more consistent, as shown in Fig. 3(a). The bladder and rectum had more consistent DSCs throughout each treatment fraction than the sigmoid and HR‐CTV, as shown in Fig. 3(b). The JRARM scores for the bladder and HR‐CTV had consistently higher medians than the rectum and sigmoid throughout the treatment fractions, as shown in Fig. 3(d). The bladder had the highest median DSC in all treatment fractions and the sigmoid had the lowest median DSC in all treatment fractions, as shown in Fig. 3(e). While the DSC results show the bladder has a lower degree of variation compared to other ROIs, the JRARM score results demonstrate that the bladder and HR‐CTV both have less motion compared to the rectum and sigmoid. The JRARM score and DSC results taken together demonstrate the bladder has a lower degree of variation compared to other ROIs, while the sigmoid has the greatest motion. Figures 3(c) and 3(f) show the variation in the RMSD metric for a comparison of volume and distance‐based metrics to compare with the JRARM score.

**Table 2:** Averages and standard deviations for DSC and JRARM score calculated from Eq. (1) for each HDR fraction to show each ROI's contour similarity from reference fraction 1 (HDR i‐1 where i=2–5)

*ROI*	*Parameter*	*HDR 2‐1*	*HDR 3‐1*	*HDR 4‐1*	*HDR 5‐1*
Bladder	DSC	0.6±0.1	0.7±0.1	0.6±0.1	0.7±0.1
	JRARM	69.0±12.4	71.6±13.2	69.4±11.7	72.8±7.1
Rectum	DSC	0.5±0.2	0.5±0.2	0.5±0.1	0.5±0.1
	JRARM	62.1±12.6	63.6±11.8	64.8±9.5	63.5±11.6
Sigmoid	DSC	0.3±0.2	0.3±0.2	0.4±0.2	0.3±0.2
	JRARM	55.7±13.6	62.5±13.1	62.3±12.5	59.5±14.1
HR‐CTV	DSC	0.5±0.2	0.4±0.2	0.5±0.2	0.4±0.2
	JRARM	71.5±9.3	69.9±10.0	67.7±13.8	70.4±11.1

**Figure 4:**
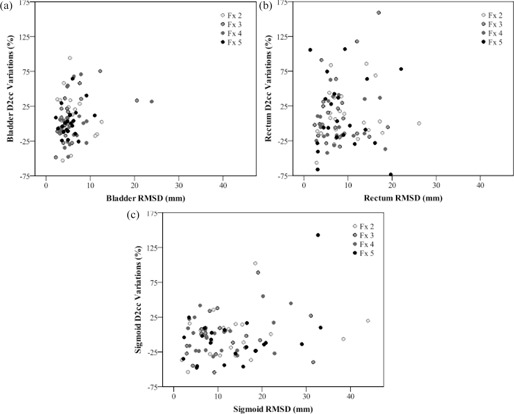
Scatter plots describing the correlation between similarity (RMSD) and dose variations (${D2}_{2\text{cc}}$) for all fractions for each patient for the (a) bladder, (b) rectum, and (c) sigmoid for the adaptive daily plan.

Physical dose variations in $D_{2\text{cc}}$ were calculated for OARs for the ADP and SP. The ADP resulted in $D_{2\text{cc}}$ variations of 3% for the bladder, 6% for the rectum, and 4% for the sigmoid. The ADP dose variations for OARs are a result of dose optimization to provide a high dose to the target while sparing OARs. The SP resulted in $D_{2\text{cc}}$ variations of 8% for the bladder, 5% for the rectum, and 4% for the sigmoid, where the dose variations to OARs were calculated based on the CT scans from the ADP. Linear regression analysis was performed to study the relationship between interfractional organ variations and dose variations from both the ADP and SP. Significant results for this relationship were found for the bladder and sigmoid. RVD, MSD, and RMSD metrics had a significant relationship with variation in $D_{2\text{cc}}$ for the bladder (p=0.000,p=0.014, and p=0.025, respectively) and sigmoid (p=0.000,p=0.032, and p=0.013, respectively). The single plan also yielded significant relationships between both volumetric and distance metrics and dose variations in the bladder (RVDp=0.000,JRARMp=0.038,ASDp=0.019,MSDp=0.001,RMSDp=0.000), rectum (RVDp=0.000, RMSD p=0.041), and sigmoid (RVDp=0.001). The RMSD metric was chosen to depict the correlation between organ similarity and dose difference in Fig. 4 since it was statistically significant in many cases for the adaptive plan. The regression results show that, not only are distance metrics important for understanding the effect of interfractional organ variation on organ dose, but also volume metrics relating to organ shape.

### B. Variations in dose between single plan and adaptive daily plan

Table 3 shows the averages and standard deviations of $D_{2\text{cc}}$ and $D_{0.1\text{cc}}$ EQD2 for each OAR for both the SP and ADP. The dose for both plans in fraction 1 is the same because the first daily plan was used for the SP and then applied to subsequent fractions. All remaining fractions (2–5) resulted in a higher dose to OARs using the SP compared to the ADP. Wilcoxon signed‐rank tests found significant differences in $D_{2\text{cc}}$ for the sigmoid with p=0.015. The difference between the SP and ADP resulted in higher $D_{2\text{cc}}$ by an average and standard deviation of $4.97\pm 14.81\;{\text{Gy}}_{\alpha /\beta =3}$ for the bladder, $3.82\pm 13.77\;{\text{Gy}}_{\alpha /\beta =3}$ for the rectum, and $4.43\pm 13.34\;{\text{Gy}}_{\alpha /\beta =3}$ for the sigmoid compared to the ADP. The ADP resulted in a lower $D_{2\text{cc}}$ to OARs compared to the SP. The $\text{HR}‐\text{CTV}\;D_{90}$ in the ADP had an average and standard deviation of $10.9\pm 4.3\;{\text{Gy}}_{\alpha /\beta =10},11.3\pm 3.1\;{\text{Gy}}_{\alpha /\beta =10},10.9\pm 3.2\;{\text{Gy}}_{\alpha /\beta =10},11.3\pm 3.0\;{\text{Gy}}_{\alpha /\beta =10}$, and $10.8\pm 2.0\;{\text{Gy}}_{\alpha /\beta =10}$ for fractions 1–5, respectively.

**Figure 5:**
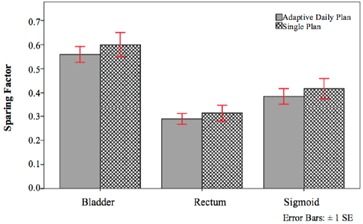
Bar graph showing sparing factors for each OAR for the adaptive daily plan and the single plan.

Sparing factors (ratio of $\text{OAR}\;D_{2\text{cc}}$ to $\text{HR}‐\text{CTV}\;D_{90}$ in EQD2),^34^, ^35^ as shown in Fig. 5, provided a metric to compare planning methods that took into account both OAR dose and HR‐CTV dose where lower values corresponded to more favorable outcomes. The sparing factors obtained for the ADP and SP were 0.56±0.15 and 0.60±0.24 for the bladder, 0.29±0.11 and 0.31±0.16 for the rectum, and 0.39±0.15 and 0.42±0.19 for the sigmoid, respectively. The sparing factors were lower for all OARs in the ADP when compared to the SP.

**Table 3:** Averages and standard deviations for DVH parameters (in ${\text{Gy}}_{\alpha /\beta =3}$) as recommended by GEC‐ESTRO II

*Dose*	*OAR*	*Plan*	*Fraction 1*	*Fraction 2*	*Fraction 3*	*Fraction 4*	*Fraction 5*	*Total*
$D_{2\text{cc}}$	Bladder	Daily	5.7±1.8	6.3±2.1	6.2±2.0	5.6±2.1	5.7±1.4	29.6±6.2
		Single	5.7±1.8	7.5±4.1	7.6±6.0	6.8±3.2	6.9±3.0	34.6±16.4
	Rectum	Daily	3.0±1.4	3.2±1.5	3.6±2.0	2.6±1.0	3.0±1.5	15.4±5.0
		Single	3.0±1.4	4.5±4.5	4.4±4.4	3.9±4.3	3.6±2.6	19.2±15.7
	Sigmoid	Daily	4.3±1.8	4.1±1.9	4.0±1.8	4.0±1.2	3.8±2.0	20.2±6.8
		Single	4.3±1.8	5.0±4.2	5.5±5.6	5.1±3.7	4.8±3.4	24.6±16.3
$D_{0.1\text{cc}}$	Bladder	Daily	10.1±4.0	10.6±4.8	9.9±3.5	9.0±3.7	8.9±2.4	48.4±12.2
		Single	10.1±4.0	12.4±7.3	13.2±12.2	11.6±6.4	11.4±5.3	58.7±31.8
	Rectum	Daily	4.8±2.6	5.2±2.5	6.3±5.0	4.4±2.2	4.9±2.8	25.6±10.0
		Single	4.8±2.6	8.5±11.3	7.9±9.4	6.7±8.6	5.9±4.5	33.7±32.6
	Sigmoid	Daily	7.3±3.7	6.8±3.2	6.6±3.1	6.8±2.3	6.5±4.0	34.0±12.5
		Single	7.3±3.7	8.4±6.8	9.7±9.2	8.9±6.5	8.6±7.9	42.9±29.0

## IV. DISCUSSION

### A. Interfractional organ and dose variations

Understanding interfractional organ motion is crucial for successful adaptive planning for cervical cancer HDR‐BT. This study investigated the interfractional variations of organ contours (HR‐CTV & OARs) during multifractionated HDR‐BT for patients. The greatest variations in JRARM scores were observed for the sigmoid, indicating that this ROI exhibits the greatest organ motion throughout the course of treatment. The substantial sigmoid motion may be due to the sigmoid being located further away from the compact organs in the pelvis. As a result of the sigmoid being distant from the compact region, it would not have as many structures surrounding it to prevent extensive motion. In addition, previous studies found high inter‐ and intraobserver variation in sigmoid delineation contributing to the variations observed.^36^, ^37^ The JRARM scores of the bladder, rectum, and HR‐CTV varied less over the treatment period compared to the sigmoid, with the bladder having the least organ motion throughout treatment fractions. The JRARM scores of the bladder and HR‐CTV showed their extent of organ motion was consistently lower compared to the other ROIs (averaging 70.70±11.24 and 69.88±11.08 over all treatment fractions, respectively). Similarly, the DSCs demonstrated that the bladder and rectum had the most consistent overlap compared to the HR‐CTV and sigmoid throughout all treatment fractions (Fig. 3(b)). The DSCs also demonstrate that the degree of bladder overlap was significantly higher compared to all ROIs in each fraction (Fig. 3(d)). The bladder was expected to vary the least due to consistent bladder volumes from consistent daily bladder filling and ease for contouring this structure. Our institution does not have any specific rectal preparation for treatment, and as a result, it is not surprising that rectal motion is appreciable.

The interfractional dose variations of the ADP were lower than those of the SP. Lang et al.^(23)^ found similar results in their study with systematic variations being small and within 6%. The adaptive plan, although accounting for organ motion, still resulted in dose variations for the same patient due to the trade‐offs between high target dose and low OAR dose. Lower variations in $\text{HR}‐\text{CTV}\;D_{90}$ compared to $\text{OAR}\;D_{2\text{cc}}$ indicates that the HR‐CTV dose remains more consistent than OAR doses and this may mainly be attributed to the fact that the brachytherapy applicator is fixed to the HR‐CTV.^(23)^ SP resulted in higher physical dose variations in $D_{2\text{cc}}$ and $D_{90}$ than ADP, strongly suggesting that ROIs receive more predictable doses from ADP due to the consideration of interfractional organ motion in the treatment plan. Furthermore, both plans’ dose variations were found to have significant relationships with both volumetric and distance metrics. This indicates that, not only may organ displacements with respect to bony anatomy have great impact on organ dose, but also organ shape and volume variations, which is the main reason we use multiple similarity measurements to evaluate adaptive planning in this study.

### B. Variations in dose between single plan and adaptive daily plan

ADP resulted in lower doses to all OARs as shown in Table 3. $D_{2\text{cc}}$ reductions were by an average and standard deviation of $4.97\pm 14.81\;{\text{Gy}}_{\alpha /\beta =3}$ for the bladder, $3.82\pm 13.77\;{\text{Gy}}_{\alpha /\beta =3}$ for the rectum, and $4.43\pm 13.34\;{\text{Gy}}_{\alpha /\beta =3}$ for the sigmoid. These dose differences between plans were found to be significantly different for the sigmoid (p=0.015). From Fig. 4, we can see that all OARs demonstrated lower sparing factors for ADP, signifying improved critical organ sparing from the adaptive planning method. It was expected that ADP would provide improved organ sparing since plan optimization is based on daily anatomical variations. The adaptive daily planning method provided improved organ sparing, while still maintaining the required dose to the HR‐CTV. The results demonstrate that adaptive daily planning can significantly reduce OAR doses when compared to the single plan. This follows what would be expected since the capability to change the dose distribution to spare normal tissues is lost when only using a single plan.

### C. Future work

In this study, rigid image registration was used to map ROI contours from CTi (i=2 to 5) to the coordinates of CT1. In our future study, deformable image registration will be explored to provide a more realistic contour mapping and dose accumulation.^38^, ^39^ CT image slice thickness^(40)^ and image modality effect on HDR treatment dosimetric uncertainties may also be investigated in the future.

## V. CONCLUSIONS

Substantial interfractional organ variations in shape and volume may occur during HDR‐BT as shown by comprehensive metrics, and the extent of this variation is significantly different among organs. It was discovered that both shape and volume interfractional organ variations were significantly related to OAR dose variations, and adaptive daily planning provides improved OAR sparing compared to single planning.

## COPYRIGHT

This work is licensed under a Creative Commons Attribution 3.0 Unported License.

## Supporting information

Supplementary Material

## References

[1] Vargo JA and Beriwal S . Image‐based brachytherapy for cervical cancer. World J Clin Oncol. 2014;5(5):921–30. 25493230 10.5306/wjco.v5.i5.921 PMC4259954

[2] Katz A and Eifel PJ . Quantification of intracavitary brachytherapy parameters and correlation with outcome in patients with carcinoma of the cervix. Int J Radiation Oncology Biol Phys. 2000;48(5):1417–25. 10.1016/s0360-3016(00)01364-x 11121642

[3] Holloway CL , Racine M‐L , Cormack RA , O'Farrell DA , Viswanathan AN . Sigmoid dose using 3D imaging in cervical‐cancer brachytherapy. Radiother Oncol. 2009;93(2):307–10. 19665244 10.1016/j.radonc.2009.06.032 PMC2867463

[4] Viswanathan AN , Beriwal S , De Los Santos J , et al. The American Brachytherapy Society consensus guidelines for locally advanced carcinoma of the cervix. Part II: High dose‐rate brachytherapy. Brachytherapy. 2012;11(1):47–52. 22265437 10.1016/j.brachy.2011.07.002 PMC3489267

[5] Chakraborty S , Patel FD , Patil VM , Oinam AS , Sharma SC . Magnitude and implications of interfraction variations in organ doses during high dose rate brachytherapy of cervix cancer: a CT based planning study. ISRN Oncol. 2014. 10.1155/2014/687365 PMC3945078 24693451

[6] Elhanafy OA , Das RK , Paliwal BR , Migahed MD , Sakr HA , Elleithy M . Anatomic variation of prescription points and treatment volume with fractionated high‐dose rate gynecological brachytherapy. J Appl Clin Med Phys. 2002;3(1):1–5. 11817998 10.1120/jacmp.v3i1.2586 PMC5724548

[7] Jones ND , Rankin J , Gaffney DK . Is simulation necessary for each high‐dose‐rate tandem and ovoid insertion in carcinoma of the cervix? Brachytherapy. 2004;3(3):120–24. 15533802 10.1016/j.brachy.2004.07.001

[8] Davidson M , Yuen J , D'Souza DP , Batchelar DL . Image‐guided cervix high‐dose‐rate brachytherapy treatment planning: Does custom computed tomography planning for each insertion provide better conformal avoidance of organs at risk? Brachytherapy. 2008;7(1):37–42. 18299111 10.1016/j.brachy.2007.12.003

[9] Potter R , Haie‐Meder C , Van Limbergen E , et al. Recommendations from gynaecological (GYN) GEC ESTRO working group (II): Concepts and terms in 3D image‐based treatment planning in cervix cancer brachytherapy— 3D dose volume parameters and aspects of 3D image‐based anatomy, radiation physics, radiobiology. Radiother Oncol. 2006;78(1):67–77. 16403584 10.1016/j.radonc.2005.11.014

[10] Assenholt MS , Petersen JB , Nielsen SK , Lindegaard JC , Tanderup K . A dose planning study on applicator guided stereotactic IMRT boost in combination with 3D MRI based brachytherapy in locally advanced cervical cancer. Acta Oncol. 2008;47(7):1337–43. 18663651 10.1080/02841860802266698

[11] Lim K , Small W , Portelance L , et al. Consensus guidelines for delineation of clinical target volume for intensity‐modulated pelvic radiotherapy for the definitive treatment of cervix cancer. Int J Radiat Oncol Biol Phys. 2011;79(2):348–55. 20472347 10.1016/j.ijrobp.2009.10.075

[12] Chan P , Dinniwell R , Haider MA , et al. Inter‐ and intrafractional tumor and organ movement in patients with cervical cancer undergoing radiotherapy: a cinematic‐MRI point‐of‐interest study. Int J Radiat Oncol Biol Phys. 2008;70(5):1507–15. 18164850 10.1016/j.ijrobp.2007.08.055

[13] Bahena JH , Martinez A , Yan D , et al. Spatial reproducibility of the ring and tandem high‐dose rate cervix applicator. Int J Radiat Oncol Biol Phys. 1998;41(1):13–19. 9588912 10.1016/s0360-3016(98)00026-1

[14] Taylor A , Powell ME . An assessment of interfractional uterine and cervical motion: implications for radiotherapy target volume definition in gynaecological cancer. Radiother Oncol. 2008;88(2):250–57. 18538873 10.1016/j.radonc.2008.04.016

[15] Lee CM , Shrieve DC , Gaffney DK . Rapid involution and mobility of carcinoma of the cervix. Int J Radiat Oncol Biol Phys. 2004;58(2):625–30. 14751536 10.1016/j.ijrobp.2003.09.060

[16] Hellebust TP , Dale E , Skjonsberg A , Olsen DR . Inter fraction variations in rectum and bladder volumes and dose distributions during high dose rate brachytherapy treatment of the uterine cervix investigated by repetitive CT‐examinations. Radiother Oncol. 2001;60(3):273–80. 11514007 10.1016/s0167-8140(01)00386-3

[17] Nesvacil N , Tanderup K , Hellebust TP , et al. A multicentre comparison of the dosimetric impact of inter‐ and intrafractional anatomical variations in fractionated cervix cancer brachytherapy. Radiother Oncol. 2013;107(1):20–25. 23602372 10.1016/j.radonc.2013.01.012 PMC3675683

[18] Jamema S V , Mahantshetty U , Tanderup K , et al. Inter‐application variation of dose and spatial location of D2cm3 volumes of OARs during MR image based cervix brachytherapy. Radiother Oncol. 2013;107(1):58–62. 23453543 10.1016/j.radonc.2013.01.011

[19] Kobayashi K , Murakami N , Wakita A , et al. Dosimetric variations due to interfraction organ deformation in cervical cancer brachytherapy. Radiother Oncol. 2015;117(3):555–58. 26316394 10.1016/j.radonc.2015.08.017

[20] Morgia M , Cuartero J , Walsh L , et al. Tumor and normal tissue dosimetry changes during MR‐guided pulsed‐dose‐rate (PDR) brachytherapy for cervical cancer. Radiother Oncol. 2013;107(1):46–51. 23540555 10.1016/j.radonc.2013.02.012

[21] Al‐Booz HF , Boiangiu I , Appleby H , et al. Sigmoid colon is an unexpected organ at risk in brachytherapy for cervix cancer. J Egypt Natl Cancer Inst. 2006;18(2):156–60. 17496941

[22] Beriwal S , Kim H , Coon D , et al. Single magnetic resonance imaging vs. magnetic resonance imaging/computed tomography planning in cervical cancer brachytherapy. Clin Oncol. 2009;21(6):483–87. 10.1016/j.clon.2009.03.007 19423307

[23] Lang S , Nesvacil N , Kirisits C , et al. Uncertainty analysis for 3D image‐based cervix cancer brachytherapy by repetitive MR imaging: assessment of DVH‐variations between two HDR fractions within one applicator insertion and their clinical relevance. Radiother Oncol. 2013;107(1):26–31. 23541645 10.1016/j.radonc.2013.02.015

[24] Mohamed S , Nielsen SK , Fokdal LU , Pedersen EM , Lindegaard JC , Tanderup K . Feasibility of applying a single treatment plan for both fractions in PDR image guided brachytherapy in cervix cancer. Radiother Oncol. 2013;107(1):32–38. 23333020 10.1016/j.radonc.2012.11.006

[25] Sumanaweera TS , Adler JR , Napel S , Glover GH . Characterization of spatial distortion in magnetic resonance imaging and its implications for stereotactic surgery. Neurosurgery. 1994;35(4):696–704. 7808613 10.1227/00006123-199410000-00016

[26] Doran SJ , Charles‐Edwards L , Reinsberg SA , Leach MO . A complete distortion correction for MR images: I. Gradient warp correction. Phys Med Biol. 2005;50(7):1343–61. 15798328 10.1088/0031-9155/50/7/001

[27] Chi A , Gao M , Sinacore J , Nguyen NP , Vali F , Alburquerque K . Single versus customized treatment planning for image‐guided high‐dose‐rate brachytherapy for cervical cancer: dosimetric comparison and predicting factor for organs at risk overdose with single plan approach. Int J Radiat Oncol Biol Phys. 2009;75(1):309–14. 19540070 10.1016/j.ijrobp.2009.03.041

[28] Van de Schoot AJ , De Boer P , Buist M , et al. Quantification of delineation errors of the gross tumor volume on magnetic resonance imaging in uterine cervical cancer using pathology data and deformation correction. Acta Oncol. 2015;54(2):224–31. 25437811 10.3109/0284186X.2014.983655

[29] Zhang M , Chen T , Kim LH , et al. Three‐dimensional dosimetric considerations from different point A definitions in cervical cancer low‐dose‐rate brachytherapy. J Contemp Brachytherapy. 2013;5(4):222–26. doi:10.5114/jcb.2013.38836 24474971 10.5114/jcb.2013.38836 PMC3899635

[30] Kouwenhoven E , Giezen M , Strikmans H . Measuring the similarity of target volume delineations independent of the number of observers. Phys Med Biol. 2009;54(9):2863–73. 19384002 10.1088/0031-9155/54/9/018

[31] Smeets D , Loecks D , Stijnen B , De Dobbelaer B , Vandermeulen D , Suetens P . Semi‐automatic level set segmentation of liver tumors combining a spiral‐scanning technique with supervised fuzzy pixel classification. Med Image Anal. 2010;14(1):13–20. 19828356 10.1016/j.media.2009.09.002

[32] Heimann T , Van Ginneken B , Styner MA , et al. Comparison and evaluation of methods for liver segmentation from CT datasets. IEEE Trans Med Imaging. 2009;28(8):1251–65. 19211338 10.1109/TMI.2009.2013851

[33] Kirisits C , Lang S , Dimopoulos J , Oechs K , Georg D , Pötter R . Uncertainties when using only one MRI‐based treatment plan for subsequent high‐dose‐rate tandem and ring applications in brachytherapy of cervix cancer. Radiother Oncol. 2006;81(3):269–75. 17126938 10.1016/j.radonc.2006.10.016

[34] Lindegaard JC , Tanderup K , Nielsen SK , Haack S , Gelineck J . MRI‐guided 3D optimization significantly improves DVH parameters of pulsed‐dose‐rate brachytherapy in locally advanced cervical cancer. Int J Radiat Oncol Biol Phys. 2008;71(3):756–64. doi:10.1016/j.ijrobp.2007.10.032 18191335 10.1016/j.ijrobp.2007.10.032

[35] Jürgenliemk‐Schulz IM , Lang S , Tanderup K , et al. Variation of treatment planning parameters (D90 HR‐CTV, D(2cc) for OAR) for cervical cancer tandem ring brachytherapy in a multicentre setting: comparison of standard planning and 3D image guided optimisation based on a joint protocol for dose–volume constraints. Radiother Oncol. 2010;94(3):339–45. 19944471 10.1016/j.radonc.2009.10.011

[36] Damato AL , Townamchai K , Albert M , et al. Dosimetric consequences of interobserver variability in delineating the organs at risk in gynecologic interstitial brachytherapy. Int J Radiat Oncol Biol Phys. 2014;89(3):674–81. 24803035 10.1016/j.ijrobp.2014.03.005 PMC4180236

[37] Saarnak AE , Boersma M , Van Bunningen BNFM , Wolterink R , Steggerda MJ . Inter‐observer variation in delineation of bladder and rectum contours for brachytherapy of cervical cancer. Radiother Oncol. 2000;56(1):37–42. 10869753 10.1016/s0167-8140(00)00185-7

[38] Varadhan R , Karangelis G , Krishnan K , Hui S . A framework for deformable image registration validation in radiotherapy clinical applications. J Appl Clin Med Phys. 2013;14(1):4066. 23318394 10.1120/jacmp.v14i1.4066 PMC3732001

[39] Yan D and Lockman D . Organ/patient geometric variation in external beam radiotherapy and its effects. Med Phys. 2001;28(4):593–602. 11339757 10.1118/1.1357224

[40] Schindel J , Zhang W , Bhatia SK , Sun W , Kim Y . Dosimetric impacts of applicator displacements and applicator reconstruction‐uncertainties on 3D image‐guided brachytherapy for cervical cancer. J Contemp Brachytherapy. 2013;5(4):250–57. 10.5114/jcb.2013.39453 PMC3899640 24474977

