# Supplementary figures and images for: A comprehensive evaluation of adaptive daily planning for cervical cancer HDR brachytherapy
Source: J Appl Clin Med Phys. 2016 Nov 8;17(6):323–33. doi: 10.1120/jacmp.v17i6.6408 (PMC5690507; doi:10.1120/jacmp.v17i6.6408)

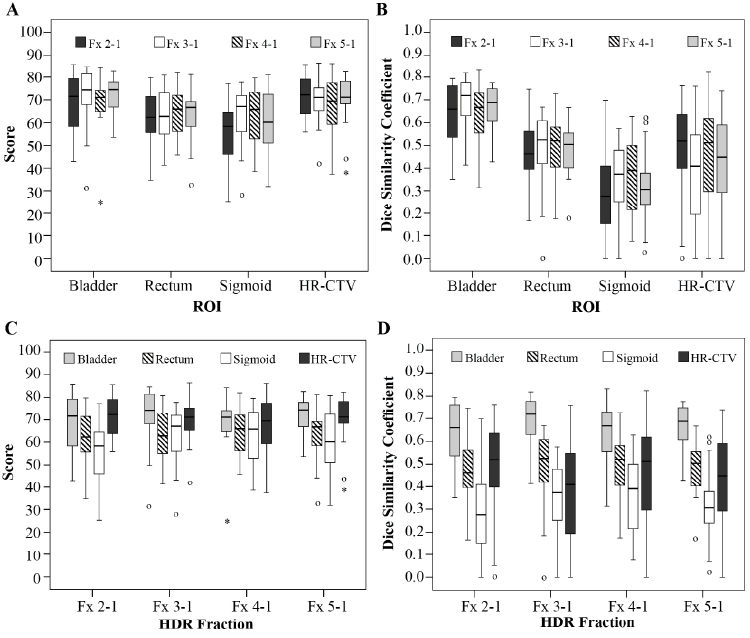

Supplement: Supplementary file 1 — Supplementary Material [file ACM2-17-323-s001.jpg]
